# Direct Observation of Intrinsic Spin–Layer Coupling and Robust Valley Polarization in Bilayer MoS_2_

**DOI:** 10.1002/smsc.202500348

**Published:** 2025-08-22

**Authors:** Yumin Sim, Je‐Ho Lee, Nguyen T. Hoang, No‐Won Park, Sang‐Kwon Lee, Maeng‐Je Seong

**Affiliations:** ^1^ Department of Physics and Center for Berry Curvature‐based New Phenomena (BeCaP) Chung‐Ang University Seoul 06974 Republic of Korea

**Keywords:** bilayer molybdenum disulfide, circularly polarized photoluminescence, degree of circular polarization, spin–layer coupling, valleytronics

## Abstract

Herein, direct evidence of intrinsic spin–layer coupling in bilayer MoS_2_ through polarization‐resolved photoluminescence measurements on fully suspended samples is presented. By eliminating substrate‐induced symmetry breaking, such as electrostatic potential gradients and unintentional strain, the intrinsic valley dynamics of bilayer MoS_2_ is isolated. The results reveal that the degree of circular polarization (DoCP) in bilayers remains significantly higher than that of monolayers across the entire temperature range. This observation cannot be explained by conventional thermal effects and instead indicates the presence of robust depolarization suppression mechanisms. In bilayer MoS_2_, spin–layer coupling not only inhibits interlayer scattering by locking spin and valley indices to individual layers but also suppression spin‐flip‐mediated intervalley scattering by constraining the spin dynamics. Together, these effects maintain a high DoCP even under conditions that would normally induce strong depolarization via rapid intervalley and interlayer scattering pathways. The observation of enhanced DoCP in bilayers, despite larger excess energy typically enhances depolarization through intervalley scattering, indicates that the polarization is intrinsically linked to spin–layer coupling. These findings establish a bilayer MoS_2_ as a compelling platform for exploring spin–valley–layer physics and advancing valleytronic applications.

## Graphical abstract

This study experimentally demonstrates finite and even enhanced circular polarization in bilayer MoS_2_, despite its centrosymmetric 2H stacking, across all temperatures. The degree of circular polarization exceeds that of monolayers, attributed to spin–layer coupling and suppression of spin‐flip processes. Substrate‐free measurements confirm intrinsic origins, offering new insights into spin and valley control in 2D semiconductors.

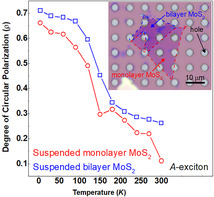

## 1 Introduction

2D transition metal dichalcogenides (TMDCs), such as monolayer MoS_2_ and WS_2_, host direct bandgaps at K and K′ points of their hexagonal Brillouin zone. In these materials, strong spin–orbit coupling (SOC) and the absence of inversion symmetry give rise to spin–valley locking, where each valley is associated with a specific spin state.^[^ ^1^, ^2^ ^]^ This unique feature allows selective excitation of carriers in specific valleys using circularly polarized light, forming the basis for valleytronics, an emerging field that encodes and manipulates information using the valley degree of freedom.^[^ ^3^, ^4^, ^5^ ^]^

Achieving a high degree of circular polarization (DoCP) is essential for valleytronic applications. Prior studies have explored various approaches to enhance valley polarization by tuning external factors such as electric fields, doping, magnetic fields, and optical engineering.^[^ ^6^, ^7^, ^8^, ^9^, ^10^, ^11^, ^12^, ^13^, ^14^, ^15^ ^]^ In addition, the Berry curvature intrinsic to TMDC band topology contributes to novel transport phenomena, such as the exciton Hall effects.^[^ ^16^, ^17^, ^18^, ^19^ ^]^ Heterostructures combining TMDCs with ferromagnetic or ferroelectric materials have also been proposed to break time‐reversal symmetry and enable ferrovalley effects, which further lift the valley degeneracy and enable nonvolatile operations.^[^ ^20^, ^21^ ^]^

While monolayer TMDCs intrinsically lack inversion symmetry and thus exhibit strong valley polarization, bilayer TMDCs with 2H stacking restore centrosymmetry and are theoretically expected to exhibit negligible DoCP.^[^ ^1^, ^22^, ^23^ ^]^ Consequently, most prior studies on TMDC‐based valley polarization have predominantly focused on monolayer system, where broken inversion symmetry enables robust optical selectivity between K and K′ valleys. However, surprisingly, measurable DoCP has been observed in bilayer TMDCs, often attributed to extrinsic symmetry‐breaking effects induced by substrate, such as strain or electrostatic potentials.^[^ ^24^, ^25^, ^26^, ^27^ ^]^ An alternative, intrinsic explanation is spin–layer coupling, in which spin states are locked to the layer index, enabling layer‐selective optical transitions even in the absence of global inversion symmetry breaking.^[^ ^4^, ^28^, ^29^, ^30^ ^]^

**Figure 1:**
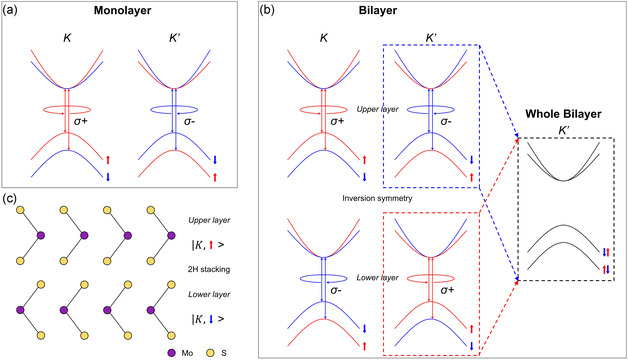
Delineation of the mechanisms of spin–valley coupling in monolayer MoS_2_ and spin–layer coupling in bilayer MoS_2_, along with the corresponding excitation and emission processes. a) Schematic presentation for the interaction between circularly polarized light and the electronic states in the K and K′ valleys of monolayer MoS_2_. b) Schematic depicting the interaction between circularly polarized light and the electronic states in the K and K′ valleys of bilayer MoS_2_. The notion ↑,↓ symbolizes the spin configurations in each valley. In the context of monolayer MoS_2_, circular polarizations of opposing nature are associated with the K and K′ valleys independently. However, in bilayer MoS_2_, opposite circular polarizations couple within the same valley but with the upper and lower layers, respectively. The “Whole Bilayer” inset highlights that the conduction and valence bands are spin‐degenerate due to inversion symmetry, with spin‐up and spin‐down states occupying identical energy levels. c) Illustration shows that carriers with spin oriented upward (downward) are confined to the upper (lower) layer within the K valley of 2H stacked bilayer MoS_2_. This visual representation underscores the distinction between spin–valley and spin–layer interactions, highlighting the nuanced electronic dynamics specified to the mono‐ and bilayer structures of MoS_2_.

This mechanism is illustrated in **Figure** **1**, which contrasts spin–valley locking in monolayer MoS_2_ and spin–layer locking in bilayer MoS_2_. In monolayers, left‐ and right‐circularly polarized light selectively coupled to opposite valleys (K and K′), each tied to specific spin orientations (Figure 1a). In contrast, the bilayer MoS_2_ exhibits a scenario where the spin state is tied to both the valley and the layer index, enabling circularly polarized light to selectively excite upper or lower layers within the same valley, depending on the spin (Figure 1b,c). In Figure 1b, although strong SOC still exists due to the presence of heavy transition metal atoms (such as Mo), the 2H stacking geometry causes the spin splitting at the respective K and K′ points but the electronic bands at $K\left(K^{\prime }\right)$ in the upper and the lower layers merge into one band for the “whole bilayer” where both layers are considered together. As a result, the electronic states at K and K′ are energy‐degenerate for spin‐up and spin‐down carriers, i.e., globally spin‐degenerate, even though each spin state is spatially separated into distinct layers due to spin–layer locking. This means that while the band energies remain degenerate, the spin texture is layer‐resolved: e.g., spin‐up states at the K valley reside predominantly in one layer (upper layer) and spin‐down states in the other (lower layer). This configuration allows circularly polarized light to selectively excite spin–layer–valley states despite the presence of overall spin degeneracy. This energy degeneracy is explicitly illustrated in the “Whole Bilayer K′” inset, where spin‐up and spin‐down states coexist at degenerate energy levels. This coupling fundamentally alters valley exciton dynamics and provides a pathway for intrinsic polarization control in bilayer systems.

Despite these theoretical proposals, most experimental investigations have been conducted on substrate‐supported samples, where unavoidable symmetry‐breaking fields may obscure the intrinsic contributions. Even if the observed DoCP is attributed to spin–layer coupling, it remains susceptible to substrate‐induced effects. This underscores the necessity of conducting measurements in substrate‐free configurations to rigorously determine the observed DoCP in bilayer MoS_2_ truly stems from intrinsic spin–layer interactions.

Our work addresses this ambiguity by employing a fully suspended bilayer MoS_2_ system, thereby eliminating extrinsic effects and enabling the unambiguous isolation of the intrinsic spin–layer coupling contribution. Therefore, we undertook controlled experiments, isolating the influence of substrate surface electronic potentials which make the symmetry breaking, and our findings experimentally support that the observed DoCP in bilayer MoS_2_ originates from spin–layer coupling. Through polarization‐resolved photoluminescence (PL) measurements across a wide temperature range, we find that bilayer MoS_2_ consistently exhibits higher DoCP than monolayer MoS_2_ under identical conditions. This enhancement cannot be explained by thermal occupation or resonant excitation effects. Instead, it arises from robust suppression of valley depolarization mechanisms, specifically, from the inhibition of spin‐flip‐mediated intervalley scattering and the energetically unfavorable nature of interlayer scattering, both of which are constrained by spin–layer locking. These findings establish bilayer MoS_2_ as a promising platform for exploring coupled spin–valley–layer physics and open new avenues for robust, long‐lived valley polarization in next‐generation valleytronic devices.

## 2 Results and Discussion

**Figure 2:**
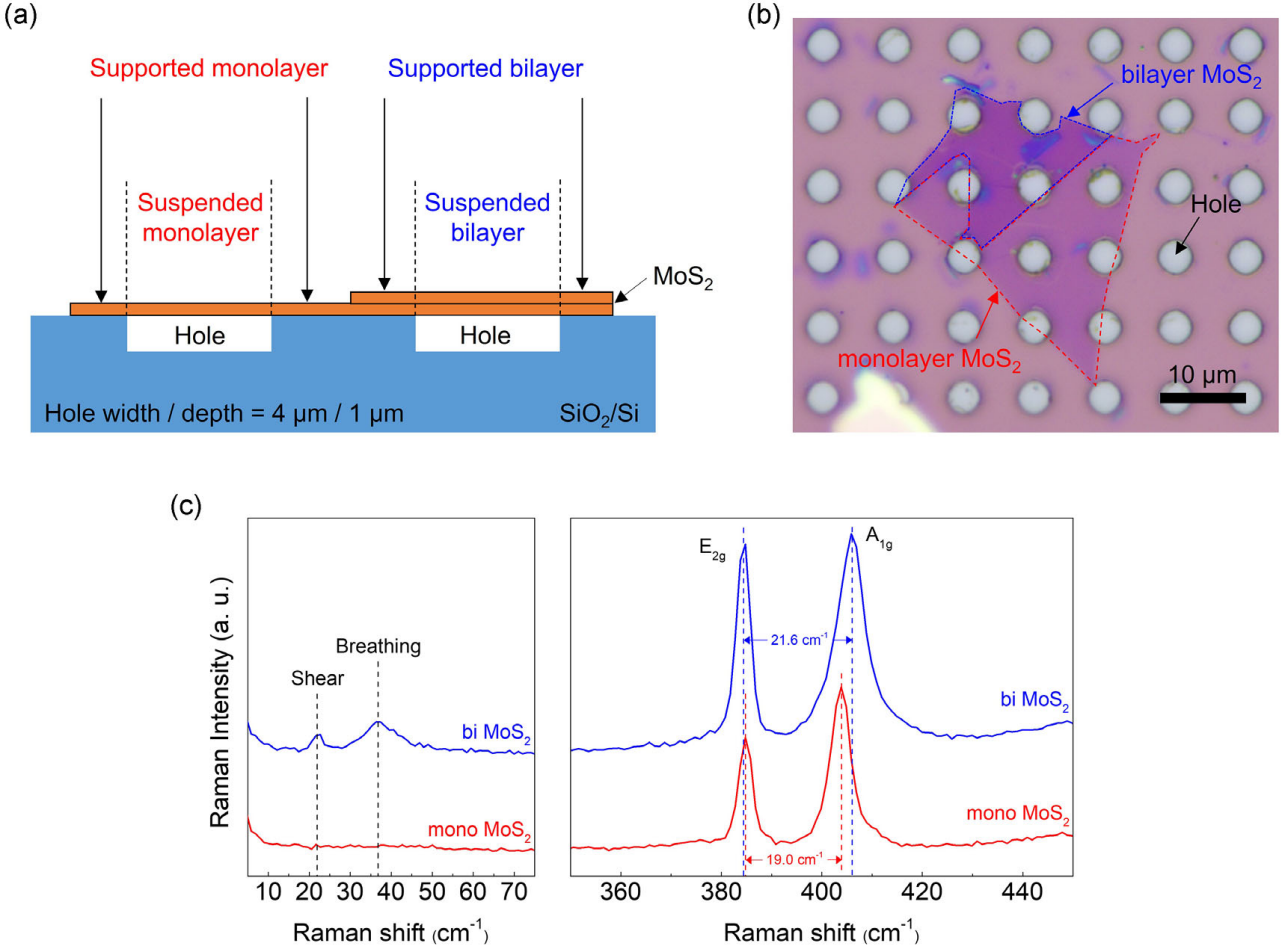
Preparation and thickness analysis of MoS_2_ samples via Raman spectroscopy. a) Schematic of the proposed MoS_2_ sample structure. By placing the sample atop substrate holes, one can negate the influence of the substrate on the analysis. b) OM image of the fabricated sample which shows a mechanically exfoliated MoS_2_ flake with distinct monolayer (red dashed lines) and bilayer (blue dashed lines) regions, transferred onto a hole‐patterned SiO_2_ substrate. White circles mark the holes in the substrate. The MoS_2_ sample can be distinctly classified into four regions: suspended monolayer, suspended bilayer, supported monolayer, and supported bilayer. The scale bar is 10 μm. c) Raman spectra of monolayer and bilayer MoS_2_. The spectrum for bilayer MoS_2_ uniquely exhibits interlayer vibrational modes (shear and breathing), which are not observed in monolayer samples. Moreover, the observed peak separations between the *E*_2g_ mode and *A*_1g_ mode are 19 cm^−1^ for the monolayer and 21.6 cm^−1^ for the bilayer, providing another clear confirmation of their respective thicknesses.

To eliminate substrate influence, we fabricated suspended MoS_2_ flake using a SiO_2_ substrate patterned with hole arrays, and its schematic illustration and optical microscopy (OM) image are shown in **Figure** **2**a and Figure 2b, respectively. As shown in Figure 2a, there are four different areas after fabricated: suspended monolayer, suspended bilayer, supported monolayer, and supported bilayer. And designed substrate hole width and depth are 4 and 1 μm, respectively. In the present experiment, a mechanically exfoliated monolayer and bilayer MoS_2_ flake was transferred to hole arrayed SiO_2_ substrate using a polymethyl methacrylate (PMMA)‐assisted wet‐transfer process.^[^ ^31^ ^]^ Microscopic image of fabricated sample (Figure 2b) shows clear monolayer sheet (red dashed lines) and bilayer sheet (blue dashed lines) on the hole arrayed SiO_2_ substrate, respectively.

We first identified the number of layers in fabricated MoS_2_ flake through Raman spectroscopy measurement which is a nondestructive and rapid technique for probing the number of layers in TMDCs. Figure 2c shows the representative Raman modes: *E*_2g_ (in‐plane vibrational mode) and *A*_1g_ (out‐of‐plane vibrational mode) at 384 and 404 cm^−1^, respectively. Raman peak position difference between these modes is 19.0 cm^−1^ in red dashed area shown in Figure 2b and 21.6 cm^−1^ in blue dashed area shown in Figure 2b, which are perfectly matched with monolayer and bilayer values reported in the literatures.^[^ ^32^ ^]^ Additionally, interlayer vibrational modes, shear and breathing, are also observed at 22 and 37 cm^−1^ in bilayer MoS_2_, respectively, while are not observed in monolayer MoS_2_.^[^ ^33^ ^]^ These Raman results show that red (blue) dashed area in the fabricated MoS_2_ flake as shown in Figure 2b is a monolayer (bilayer).

**Figure 3:**
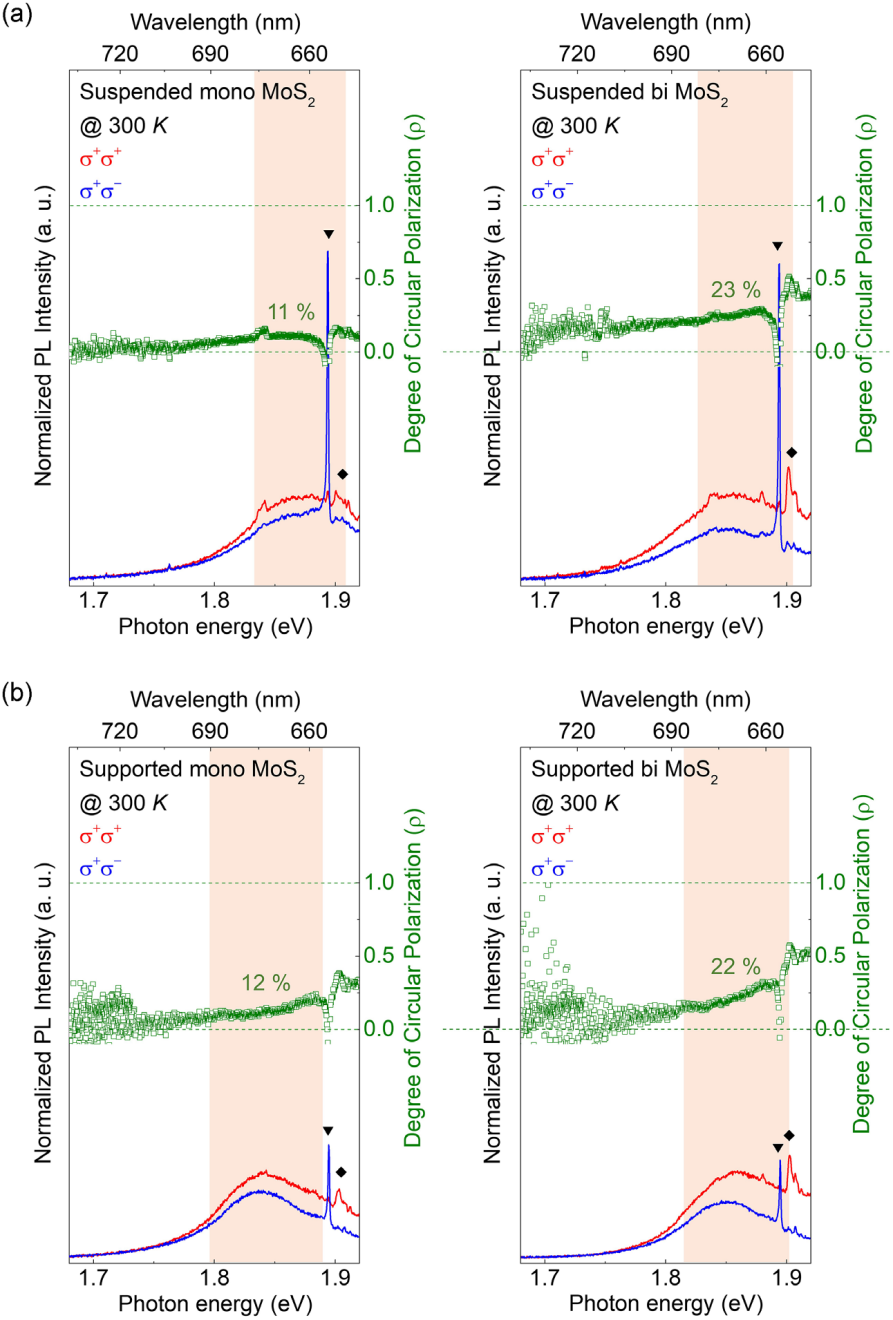
Circularly polarized PL spectra at 300 K and resulting DoCP analysis for both suspended monolayer and bilayer MoS_2_, where the red‐shaded area denotes the convolution of emission features arising from both A‐exciton and trion. The symbols ▾ and ♦ indicate the Raman signals from SiO_2_/Si substrate and MoS_2_, respectively. a) The left and right panels display the PL spectra and DoCP for suspended monolayer and bilayer MoS_2_, respectively. The DoCP is 11% for the monolayer and 23% for the bilayer. The observation of DoCP in bilayer MoS_2_, free from substrate influence, provides clear evidence that the observed DoCP originates from spin–layer coupling. b) The left and right panels similarly show the PL spectra and DoCP for supported monolayer and bilayer MoS_2_, with measured values of 12% and 22%, respectively. The comparable DoCP values in suspended and supported MoS_2_ samples indicate that the SiO_2_ substrate has minimal impact on the DoCP of both monolayer and bilayer MoS_2_.

Now, we discuss the PL spectra of suspended MoS_2_ samples where the influence of the substrate is excluded. **Figure** **3**a shows the obtained circularly polarized PL spectra at 300 K, excited by $\sigma^{+}$ radiation at 1.96 eV (633 nm), with its DoCP (ρ, inset, green). We quantify the DoCP

(1) $$\rho =\frac{I\left(\sigma^{+}\right)-I\left(\sigma^{-}\right)}{I\left(\sigma^{+}\right)+I\left(\sigma^{-}\right)}$$

determined from the circular PL intensities $I\left(\sigma^{\pm }\right)$. MoS_2_ is typically intrinsic n‐type conductivity, primarily due to sulfur vacancies and unintentional doping, which introduce excess electrons into the system. This electron‐rich environment facilitates the formation of negatively charged excitons (trions), wherein a neutral exciton binds with an additional free electron. These trions, like neutral excitons, have been reported to retain valley polarization, indicating that the spin–valley locking mechanism extends to charged excitonic complexes as well.^[^ ^34^ ^]^ Trions exhibits a binding energy of ≈30 meV, which results in a PL peak that appears roughly 30 meV lower in energy compared to the neutral A‐exciton peak.^[^ ^35^ ^]^ Consequently, the PL spectrum near 1.9 eV reflects overlapping emission from A‐exciton and trion. In principle, deconvolution using multipeak Gaussian fitting could allow quantitative separation of these features. However, under the resonant excitation condition (1.96 eV), the PL peak profile is significantly distorted by overlapping with strong Rayleigh scattering and Raman modes of both MoS_2_ and the substrate. This spectral interference leads to asymmetries and broadening in the emission line shape, preventing reliable fitting. Even if a fit was performed, the extracted parameters would be highly uncertain and potentially misleading. For these reasons, we chose to present the unprocessed spectra, preserving the full structure of the experimental data. This approach ensures transparency and avoids introducing artifacts that could obscure the intrinsic valley polarization behavior.

Although bilayer MoS_2_ exhibits an indirect bandgap with the valence band maximum shifting from the K to the Γ point, A‐exciton PL emission from the direct K−K transition remains observable.^[^ ^36^ ^]^ This is due to thermal population at the K point and the significantly higher radiative efficiency of direct transitions compared to phonon‐assisted indirect ones. While the shift in band extrema reduces the strength of valley‐selective optical responses relative to monolayers, the residual K point activity still contributes measurably to the PL signal and supports interpretation within the K‐valley framework, though with caution.

**Figure 4:**
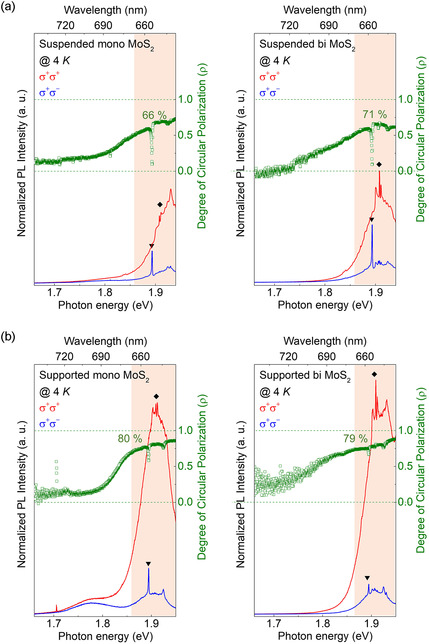
Circularly polarized PL spectra measured at 4 K and the corresponding DoCP, where the red‐shaded area denotes the convolution of emission features arising from both A‐exciton and trion with ▾ (♦) marking the Raman signals from SiO_2_ (MoS_2_). a) The left and right images display the PL spectra and DoCP for suspended monolayer and bilayer MoS_2_, respectively. The DoCP significantly increased to 66% for the monolayer and 71% for the bilayer. This increase in DoCP can be attributed to the blueshift of the exciton PL peak energy, resulting in closer resonant excitation condition and thereby reducing excess energy. b) For supported monolayer and bilayer MoS_2_, the DoCP values are 80% and 79%, respectively, indicating an average increase of 8% over the suspended MoS_2_ samples, Unlike at 300 K, the substrate appears to influence the DoCP, which can be attributed to the different thermal expansion coefficients between SiO_2_ and MoS_2_.

The sharp features observed in the PL spectra of Figure 3 and **Figure** **4** (marked with the symbols ▾ and ♦) originate from first‐ and second‐order Raman modes of MoS_2_ and substrate, which are inevitably captured under our measurement conditions, specifically, the backscattering geometry and high spectral resolution employed during resonant excitation near the A‐exciton transition.^[^ ^37^ ^]^ Such Raman features are commonly observed in TMDCs PL spectra under resonant excitation and do not interfere with the analysis the fidelity and reproducibility of the raw experimental data. While we acknowledge that removing or masking the Raman peaks could enhance visual clarity, doing so would require postprocessing or interpolation that may introduce ambiguity or distort the original signal. Therefore, for the sake of transparency and data integrity, we present the unaltered spectra as measured.

As shown in the left panel of Figure 3a, ρ of ≈11% for the suspended monolayer MoS_2_, which can be attributed to the inversion asymmetry as discussed already. Interestingly, suspended bilayer MoS_2_, where the inversion symmetry is restored, also shows ρ of ≈23% at room temperature. This finding serves as compelling evidence that the observed DoCP in suspended bilayer MoS_2_ primarily arises from the intrinsic spin–layer locking effect, rather than extrinsic symmetry breaking effects introduced by the substrate. In addition, although bilayer MoS_2_ is further off‐resonance compared to monolayer MoS_2_, its DoCP is still twice as high. In general, the reduction in the DoCP observed in monolayer MoS_2_ is attributed to the transition of electrons, excited in the valence band with energy exceeding the bandgap, to another valley. The surplus excitation energy facilitates intervalley scattering, reducing the DoCP.^[^ ^3^, ^4^ ^]^ Notably, the higher DoCP observed in bilayer MoS_2_, despite its larger excess energy as compared to monolayer, suggests that excess energy cannot account for the observed polarization behavior. A more detailed discussion of this point will be provided in a subsequent section. Remarkably, the substantial enhancement in DoCP observed in bilayer MoS_2_ under identical experimental conditions provides strong evidence that the dominant origin of the polarization in bilayer MoS_2_ arises from the spin–layer locking effect.

To investigate the influence of substrate factors, such as surface electrostatic potential that could induce structural symmetry breaking in MoS_2_, the same circularly polarization‐resolved PL and DoCP measurements, as conducted on suspended MoS_2_, were performed on the supported MoS_2_ flakes, and the results are presented in Figure 3b. As shown in Figure 3b, the DoCP for supported monolayer MoS_2_ and supported bilayer MoS_2_ was 12% and 22%, respectively, which are nearly identical to those measured in suspended counterparts. These findings imply that the substrate effect on DoCP is negligible, supporting the idea that the observed DoCP in bilayer MoS_2_ is attributed to the spin–layer locking effect.

Figure 4a presents the PL and DoCP results of suspended MoS_2_ samples observed at 4 K. Generally, for TMDC monolayers, a decrease in temperature results in a blueshift of the A‐exciton PL peak position, bringing it closer to the laser excitation energy and thereby approaching resonant conditions by reducing the excess energy, the difference between the excitation laser energy and the exciton bandgap. This reduction in excess energy diminishes the likelihood of intervalley scattering of excited electrons, naturally leading to an increase in DoCP. As evidenced in the case of suspended monolayer MoS_2_ shown on the left side of Figure 4a, the exciton PL peak exhibits a blueshift while the DoCP increases sixfold to 66% compared to the value measured at 300 K. This phenomenon can similarly apply to the spin–layer locking effect. As the temperature decreases, bilayer MoS_2_ also experiences a reduction in excess energy through the blueshift of the exciton PL peak, which is expected to decrease the possibility of excited electron intervalley scattering and interlayer scattering, naturally leading to an increase in DoCP. As seen on the right side of Figure 4a, suspended bilayer MoS_2_ at 4 K also shows a blueshift in the exciton PL peak along with an increased DoCP reaching 71%. Furthermore, similar to the observations at 300 K, the DoCP of bilayer MoS_2_ remains relatively higher than that of monolayer MoS_2_ at 4 K, indicating that electron intervalley and interlayer scattering is effectively suppressed at lower temperatures. In addition to the improved resonance conditions, the suppression of phonon‐assisted intervalley scattering at cryogenic temperatures further contributes to the enhancement of DoCP. As the phonon population decreases with cooling, the provability of phonon‐mediated momentum‐ and spin‐flip scattering events between K and K′ valleys is significantly reduced, leading to more robust preservation of valley polarization.^[^ ^38^, ^39^ ^]^ These two mechanisms, the approach to resonant excitation and the suppression of phonon‐driven depolarization, jointly account for the pronounced increase in circular polarization observed in both monolayer and bilayer configurations at low temperatures.

To further understand the effect of the substrate at 4 K, like the experiments conducted at 300 K, measurements of circularly polarized PL and DoCP were performed using supported MoS_2_ samples, with the results detailed in Figure 4b. Unlike at 300 K, where the influence of the substrate was considered negligible, at 4 K, the DoCP values for monolayer MoS_2_ and bilayer MoS_2_ were observed to increase to 80% and 79% respectively, indicating an overall increase in DoCP compared to the values at 300 K. This phenomenon can be attributed to the difference in thermal expansion coefficients between the SiO_2_ substrate and MoS_2_.^[^ ^38^ ^]^ Generally, as the temperature decreases from 300 to 4 K, structural contractions occur. MoS_2_, having a higher thermal expansion coefficient compared to the SiO_2_ substrate, should experience greater contraction. However, due to the adhesive forces between the substrate and MoS_2_, a structural strain is induced in MoS_2_. This strain leads to symmetry breaking, which ultimately results in an increase in DoCP for both monolayer and bilayer MoS_2_, a consequence of strain‐induced symmetry breaking. Such strain manifestation may also contribute to physical defects in the MoS_2_ flake, as indirectly evidenced in the left panel of Figure 4b. At 4 K, the ≈1.76 eV PL in supported monolayer MoS_2_ is likely to originate from defect‐bound excitons.^[^ ^40^ ^]^ Its absence in suspended sample and at 300 K supports the interpretation that it arises from substrate‐induced defects, which become more prominent at low temperature due to strain relaxation and enhanced carrier trapping.

**Figure 5:**
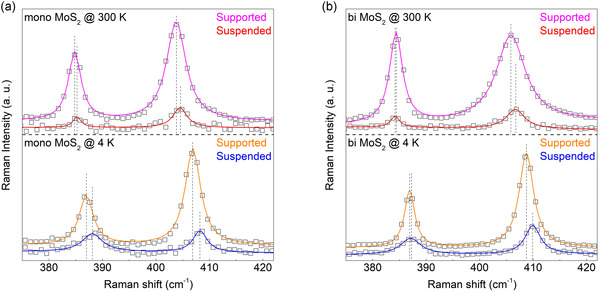
Analysis of Raman shift in MoS_2_ in relation to substrate effects and temperature changes. a) The figure demonstrates the shift in Raman peak positions of monolayer MoS_2_ measured at 300 and 4 K, differentiating between samples with and without an SiO_2_ substrate. A more significant shift in MoS_2_ Raman peak position is observed at 4 K than 300 K. This is because the strain effect, resulting from them mismatch in thermal expansion coefficients between SiO_2_ and MoS_2_, becomes more pronounced as the temperature decreases. This strain effect induces a structural distortion in MoS_2_, impacting its vibrational modes. b) Similar to the monolayer case, the figure shows the Raman peak position shifts of bilayer MoS_2_ with and without SiO_2_ substrate at 300 and 4 K. Consistent with the monolayer observations, the extent of the Raman peak shifts is more pronounced at 4 K. These Raman shifts corroborate the findings presented in **Figure 4**, where an increased DoCP in supported MoS_2_ at 4 K, relative to suspended samples, is attributed to strain‐induced structural symmetry breaking in MoS_2_. This symmetry breaking is a result of the mismatch in thermal expansion coefficients between substrate and MoS_2_, confirming the influence of substrate‐induced strain on the material's structural and electronic properties.

The presence of strain can be easily verified through Raman spectroscopy. Typically, the application of uniaxial strain to MoS_2_ layers results in a redshift of the Raman peak positions.^[^ ^41^, ^42^ ^]^ To confirm the occurrence of strain as temperature decreases, Raman spectroscopy measurements were conducted on both suspended and supported MoS_2_ samples at 300 and 4 K, with the measured spectra displayed in **Figure** **5**. The exact peak positions of the *E*_2g_ and *A*_1g_ modes in all spectra were determined through Lorentzian peak fitting, allowing for the observation of the temperature‐dependent differences in *E*_2g_ and *A*_1g_ mode peak positions for monolayer and bilayer MoS_2_ in Figure 5a,b, respectively. A more significant difference (redshift) in Raman peak positions between suspended and supported MoS_2_ is clearly observed at 4 K compared to 300 K. This indicates a stronger substrate‐induced strain effect at 4 K, supporting the increase in DoCP observed in Figure 4 because of strain‐induced symmetry breaking. Similar observations can be made for bilayer MoS_2_ as shown in Figure 5b, but the redshift in Raman peak positions for bilayer MoS_2_ is smaller than for monolayer MoS_2_, suggesting a relatively smaller strain. This difference is also reflected in the rate of DoCP increase from 300 to 4 K, where the increases for monolayer and bilayer MoS_2_ are 14% and 8%, respectively, indicating a relatively stronger strain effect in monolayer MoS_2_. This observation is consistent with the Raman spectroscopy results in Figure 5, suggesting a larger impact of strain on the monolayer MoS_2_ compared to the bilayer MoS_2_.

**Figure 6:**
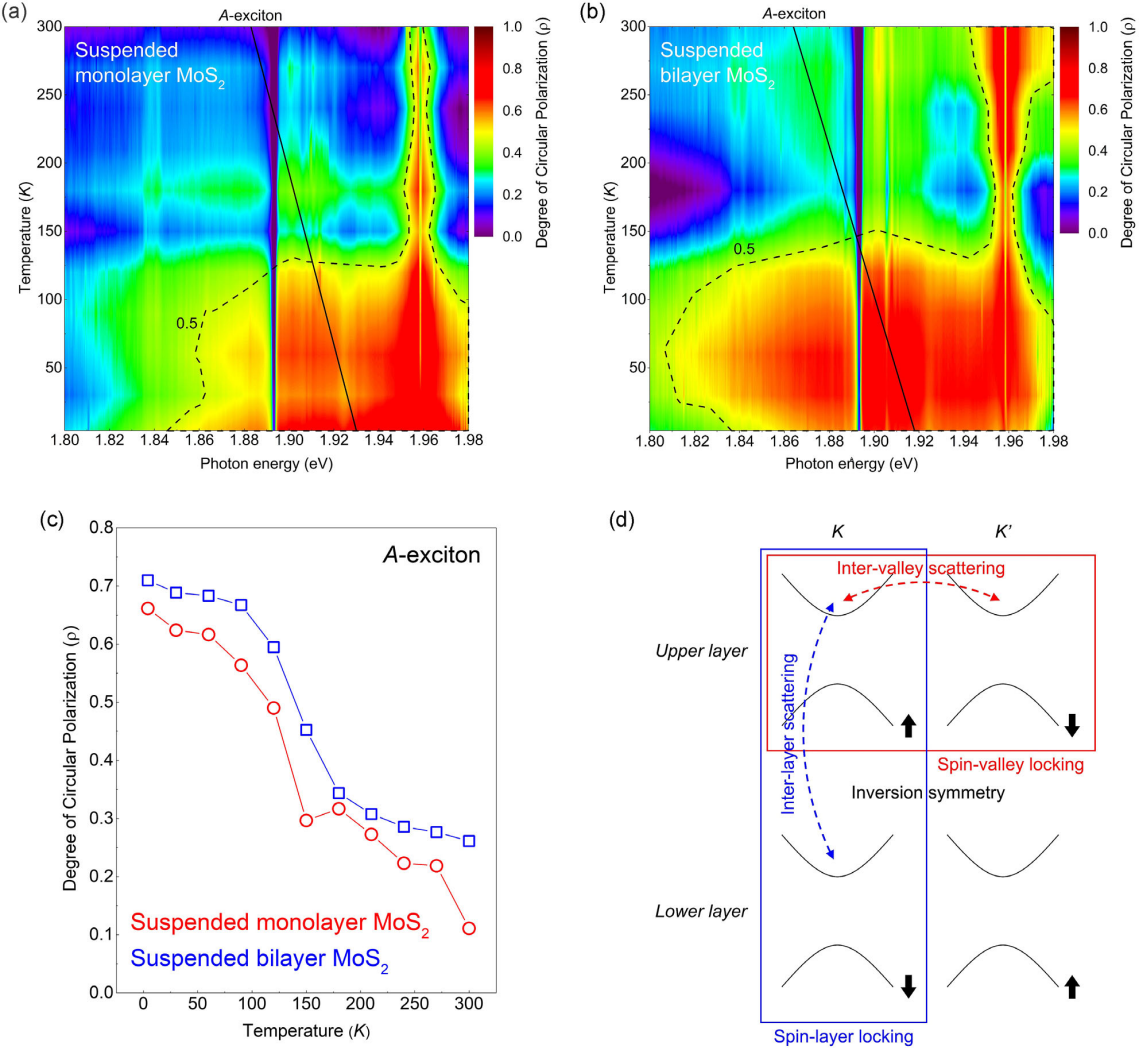
Temperature‐ and energy‐resolved DoCP map for suspended a) monolayer and b) bilayer MoS_2_. In contrast to the monolayer, the bilayer maintains a high DoCP across a wide temperature range. c) Temperature‐dependent DoCP extracted at the A‐exciton peak position (black solid line in Figure 6a,b) for suspended monolayer (red circles) and bilayer (blue squares) MoS_2_. The bilayer consistently exhibits higher DoCP at all temperatures. d) Schematic illustration of spin–valley (red panel) and spin–layer (blue panel) coupling in bilayer MoS_2_. Thermal excitation promotes intervalley (red dashed arrow) and interlayer (blue dashed arrow) scattering, leading to depolarization.

One of the most noteworthy aspects of our experimental results is that the DoCP is consistently higher in bilayer MoS_2_ than in monolayer MoS_2_ at both 300 and 4 K. To investigate this behavior in more detail, we conducted temperature‐dependent valley polarization measurements, as summarized in **Figure** **6**. Figure 6a,b shows the temperature dependence of DoCP in suspended monolayer and bilayer MoS_2_, respectively. Comparison of the area above the dotted guideline at DoCP = 0.5 reveals that bilayer MoS_2_ maintains a significantly broader range of high DoCP values compared to the monolayer, indicating that bilayer MoS_2_ exhibits stronger valley polarization across the entire temperature range. This trend is further quantified in Figure 6c, where the temperature‐dependent DoCP at the A‐exciton peak is compared between the two systems. On average, the bilayer MoS_2_ shows an 8% higher DoCP than the monolayer across all temperatures, confirming that the observed enhancement is not limited to specific thermal conditions.

Conventionally, it has been assumed that due to the restored inversion symmetry in bilayer MoS_2_, the DoCP should either be negligible or substantially lower than in monolayer systems, However, our results demonstrate the opposite trend, with the bilayer MoS_2_ maintaining a higher DoCP at all measured temperatures. Similar observations have been reported in the bilayer WS_2_, where the DoCP was found to be 60% at 300 K and 95% at 4 K, compared to 10% and 40%, respectively, in monolayer WS_2_.^[^ ^43^ ^]^ Although the absolute values differ due to material‐specific SOC strengths and exciton dynamics, the overall trend of enhanced DoCP in bilayers is consistent with our results in MoS_2_. This agreement supports the general picture that spin–layer locking in bilayer TMDCs contributes to stronger valley polarization. These results challenge conventional understanding and call for a deeper examination of the underlying depolarization mechanisms.

While the observation of enhanced DoCP in bilayer MoS_2_ is experimentally evident, a complete theoretical framework that quantitatively accounts for this enhancement over monolayer is currently lacking. Nonetheless, previous theoretical studies have shown that spin–layer coupling in 2H‐stacked bilayers can give rise to finite circular polarization, even in the presence of inversion symmetry, by enabling layer‐resolved optical selection rules.^[^ ^28^ ^]^

Generally, DoCP degradation arises from depolarization processes such as exciton recombination, intervalley scattering, and interlayer transitions. According to the relation $P=P_{0}/\left(1+\frac{2\tau }{\tau_{k}}\right)$, where P is the circular polarization, $P_{0}$ is the theoretical maximum polarization, τ is the exciton lifetime, and $\tau_{k}$ is the valley lifetime, one would expect bilayer MoS_2_ to exhibit lower DoCP due to its longer exciton lifetime and shorter valley lifetime compared to the monolayer.^[^ ^2^ ^]^ This discrepancy implies the existence of additional mechanisms that actively contribute to sustaining valley polarization.

In monolayer MoS_2_, valley depolarization occurs primarily through intralayer intervalley scattering $\left(K_{↑}↔K\prime_{↓}\right)$, which requires simultaneous valley switching and spin‐flip. In bilayer systems, however, two distinct depolarization channels are present, as illustrated in Figure 6d: intralayer intervalley scattering (red dotted arrow), similar to the monolayer case, and interlayer scattering unique to the bilayer structure (blue dotted arrow).

For intralayer intervalley scattering, phonon‐assisted momentum transfer enables valley switching, but a concurrent spin‐flip is required for depolarization, making spin relaxation the key limiting factor in intralayer intervalley scattering. The spin‐flip processes are generally governed by three primary mechanisms: D'yakonov–Perel (DP),^[^ ^44^ ^]^ Elliot–Yafet (EY),^[^ ^45^ ^]^ and Bir–Aronov–Pikus (BAP).^[^ ^46^ ^]^ The DP mechanism, which depends on Rashba‐type SOC induced by electric field or asymmetric environments, is suppressed in suspended samples due to the absence of substrate‐induced field or strain. Therefore, the DP mechanism can be reasonably excluded from further consideration in our analysis, as it is expected to be negligible under the present experimental conditions. The EY mechanism involves spin admixture, where the electron Bloch state is expressed as $\left|\Psi_{\vec{k}\cdot n}\rangle =a_{\vec{k}}|↑\rangle +b_{\vec{k}}|↓\right\rangle$, and the degree of spin mixing is given by |bk→ak→|=LΔ, with L being the SOC matrix element and *Δ* the energy gap between bands.^[^ ^47^ ^]^ Stronger SOC leads to greater spin admixture and, correspondingly, faster spin relaxation: 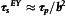, where 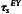 is the spin relaxation time and $\tau_{p}$ is the momentum relaxation time. Due to inversion symmetry in bilayers and spin–layer locking, SOC is weaker,^[^ ^48^ ^]^ and spin admixture is reduced, leading to suppressed spin‐flip events and enhanced DoCP. In the BAP mechanism, spin‐flips occur via exchange interaction between electrons and holes, quantified by the exchange value $J_{\mathrm{ex}}\propto \int {\left|\Psi_{e}\left(\vec{r}\right)\right|}^{2}{\left|\Psi_{h}\left(\vec{r}\right)\right|}^{2}d\vec{r}$. In other words, $J_{\mathrm{ex}}$ scales with the spatial overlap between the electron and hole wavefunctions. Since the bilayer MoS_2_ has a lower exciton binding energy (≈0.424 eV) compared to monolayers (≈0.897 eV),^[^ ^49^ ^]^ the wavefunction overlap is reduced, further suppressing spin‐flip via the BAP mechanism. Together, this analysis indicates that intralayer spin‐flip processes are more effectively suppressed in bilayer MoS_2_ due to weakened EY and BAP mechanisms, leading to stronger retention of valley polarization.

In terms of interlayer scattering, spin–layer coupling plays a pivotal role. In bilayer MoS_2_, spin orientation is intrinsically coupled to both the valley and the layer index. Thus, valley depolarization via interlayer processes requires a spin‐flip and a layer change to occur simultaneously. Interlayer scattering in bilayer MoS_2_ generally demands higher energy compared to intervalley scattering, primarily due to the additional energy to facilitate transition between layers.^[^ ^43^ ^]^ This multifaceted transition is symmetry‐restricted and energetically unfavorable, effectively narrowing the available depolarization pathways and preserving valley polarization beyond the intrinsic valley lifetime.

In addition to the suppression of interlayer and spin‐flip scattering, the influence of structural defects on valley polarization is also expected to be minimal in our system. The use of mechanically exfoliated high‐quality bilayer MoS_**2**_ and a contamination‐reducing wet transfer process minimizes the introduction of structural imperfections. Moreover, the suspended geometry eliminates substrate‐induced strain and charge trapping, which often activate defect‐related valley depolarization. This interpretation is supported by the absence of defect‐bound exciton emission around ≈1.76 eV in the suspended bilayer spectra, even at 4 K as shown in Figure 4.

Furthermore, while unintentional doping can influence valley polarization, its contribution is expected to be minimal in our suspended samples. This is supported by Raman *A*_1g_ mode analysis shown in Figure 5, where the observed linewidth narrowing in suspended regions indicates reduced electron–phonon coupling and lower residual carrier concentration.^[^ ^50^ ^]^ Specifically, the *A*_1g_ linewidth decreased from 4.4 cm^−1^ in supported to 3.5 cm^−1^ in suspended samples, on average. We also note that potential in‐plane sliding of the top layer in the bilayer MoS_2_, which could break inversion symmetry, is unlikely to occur in our samples. The flakes were exfoliated from bulk 2H‐phase crystals and transferred without lateral strain, minimizing interlayer displacement. Additionally, Raman spectra show no evidence of stacking disorder as shown in Figure 2, indicating that the structural symmetry is preserved. Therefore, these findings collectively indicate that the observed enhancement in DoCP originates primarily from intrinsic spin–layer coupling, rather than defect‐related effects.

While our analysis offers a qualitative understanding of the high DoCP observed in the bilayer MoS_2_, further studies are required to quantitatively characterize the interplay between spin–layer coupling, exciton dynamics, and scattering mechanisms. In particular, theoretical modeling is still needed to fully account for the relative magnitude of DoCP in bilayer systems and its dependence on spin, valley, and interlayer scattering processes. In this context, while our study focuses on intrinsic spin–layer coupling under zero external field, recent theoretical studies suggest that applying a vertical electric or magnetic field can further tune spin and valley polarization by breaking inversion or time‐reversal symmetry.^[^ ^51^, ^52^ ^]^ Investigating such field‐induced control in bilayer MoS_2_ would not only deepen the understanding of spin–valley physics but also open pathways for device‐level applications in valleytronics and magnetoelectric systems.

Moreover, due to time‐reversal symmetry in 2H‐stacked bilayer MoS_2_, $\sigma^{-}$ circularly polarized light excites both the lower layer at the K valley and the upper layer at the K′ valley. Since these two transitions are energetically degenerate and optically equivalent, standard far‐field PL measurements cannot distinguish their layer or valley origin. Therefore, resolving such contributions would require external symmetry‐breaking fields or layer‐sensitive techniques such as second‐harmonic generation microscopy, which are beyond the scope of the present study. Nonetheless, these findings highlight the potential of bilayer MoS_2_ as a promising platform for valleytronics applications, where long‐lived and robust valley polarization is essential.

## 3 Conclusion

In this study, we demonstrated that significant valley‐selective circular polarization persists in bilayer MoS_2_ even under fully suspended conditions, thereby eliminating the influence of substrate‐induced symmetry breaking. This unambiguously supports spin–layer coupling as the intrinsic origin of valley polarization in the bilayer system. Furthermore, the DoCP of bilayer MoS_2_ remains consistently higher than that of monolayer MoS_2_ across all measured temperatures. This temperature‐dependent enhancement cannot be attributed to thermal occupation or resonant excitation effects but rather indicates that interlayer‐coupled spin–valley–layer locking effectively suppressed depolarization channels, including intervalley and interlayer scattering. These findings clarify the microscopic origin of circular polarization in bilayer TMDCs and underscore the robustness of spin–layer coupling as a key mechanism for preserving valley information, offering a compelling route toward valleytronic device applications.

## 4 Experimental Section

4.1

4.1.1

##### Sample Preparation

Monolayer and bilayer MoS_2_ flakes were mechanically exfoliated from bulk MoS_2_ crystals (HQ Graphene Co.) using standard Scotch tape methods and initially deposited onto SiO_2_/Si substrates. Selected flakes were subsequently transferred onto prepatterned hole‐arrayed SiO_2_/Si substrate via a PMMA‐assisted wet‐transfer process.

To prepare for the transfer, a layer of PMMA was spin‐coated onto the exfoliated sample, beginning with a prespinning step at 500 rpm for 10 s, followed by spin coating at 4000 rpm for 60 s. The sample was then soft baked at 120 °C for 2 min. Delamination from the SiO_2_ substrate was achieved by immersing the PMMA‐coated sample in 5% hydrofluoric acid (HF) solution for 1 min, followed by rinses in deionized (DI) water several times to remove residual HF. The detached PMMA/MoS_2_ stack was transferred onto a hole‐arrayed substrate that had been pretreated with oxygen plasma (20 sccm, 30 s) to improve surface adhesion. After transfer, the sample was left to dry in ambient conditions for over 24 h. PMMA removal was performed via sequential solvent cleaning: immersion in acetone (13 min), isopropanol (15 min), and DI water (20 min). The final sample was dried and baked on a hot plate at 120 °C for 2 min to complete the transfer process.

##### Characterization

Raman and PL spectra at room temperature were measured using a 633 nm laser, focused on the sample through a 50× objective lens, resulting in a laser spot size of ≈1 μm. Scattered light from the samples was analyzed using a single grating spectrometer (Princeton, SP‐2500i) with a focal length of 50 cm and detected by a liquid nitrogen‐cooled CCD detector (Princeton, Spec‐10 IR enhanced). All measured spectra were calibrated using a standard Neon calibration lamp (Newport, 6032). For Raman and PL spectra at cryogenic temperatures, samples were placed inside a vibration‐free cryostat (Montana Instrument Co., Cryostation s50), and measurements were conducted in the same manner as at room temperature. Temperature‐dependent PL spectral images were acquired by adjusting the temperature from 4 to 300 K in 30 K intervals, with a 20 min stabilization period for the sample after each temperature change. Circularly polarized PL spectra were achieved using two quarter‐wave plates: the first to convert the incident light into right circular polarization ($\sigma^{+}$) and the second to select between right ($\sigma^{+}$) and left ($\sigma^{-}$) circular polarization, thereby creating parallel ($\sigma^{+}\sigma^{+}$) and cross ($\sigma^{+}\sigma^{-}$) circularly polarized configurations.

## Conflict of Interest

The authors declare no conflict of interest.

## Author Contributions

**Yumin Sim** and **Je‐Ho Lee** conceived and designed the experiment. **Yumin Sim** performed experiments. **Yumin Sim** and **Nguyen T. Hoang** analyzed the data. **Yumin Sim** and **No‐Won Park** prepared samples. **Sang‐Kwon Lee** and **Maeng‐Je Seong** supervised the research. **Yumin Sim** drafted the manuscript. All authors contributed to review and editing. **Yumin Sim** and **Je‐Ho Lee** contributed equally to this work.

## Data Availability

The data that support the findings of this study are available from the corresponding author upon reasonable request.
